# Assessing household lifestyle exposures from consumer purchases, the My Purchases cohort

**DOI:** 10.1038/s41598-023-47534-6

**Published:** 2023-12-07

**Authors:** Frederik T. Møller, Thor Grønborg Junker, Kathrine Kold Sørensen, Caroline Eves, Jan Wohlfahrt, Joakim Dillner, Christian Torp-Pedersen, Bartlomiej Wilkowski, Steven Chong, Tune H. Pers, Victor Yakimov, Heimo Müller, Steen Ethelberg, Mads Melbye

**Affiliations:** 1 Department of Infectious Disease Epidemiology and Prevention, Statens Serum Institut, Copenhagen, Denmark; 2 Department of Epidemiology Research, Statens Serum Institut, Copenhagen, Denmark; 3 Department of Cardiology, North Zealand Hospital, Hillerød, Denmark; 4 Department of Public Health, University of Copenhagen, Copenhagen, Denmark; 5 Danish Cancer Society Research Center, Copenhagen, Denmark; 6 Department of Clinical Science, Intervention and Technology, Karolinska Institutet, Stockholm, Sweden; 7 Department for Digital Infrastructure, Statens Serum Institut, Copenhagen, Denmark; 8 The Novo Nordisk Foundation, Center for Basic Metabolic Research, University of Copenhagen, Copenhagen, Denmark; 9 Department for Congenital Disorders, Statens Serum Institut, Copenhagen, Denmark; 10 Diagnostic and Research Center for Molecular BioMedicine, Medical University of Graz, Graz, Austria; 11 Department of Public Health, Global Health Section, University of Copenhagen, Copenhagen, Denmark

**Keywords:** Epidemiology, Lifestyle modification, Preventive medicine, Nutrition

## Abstract

Consumer purchase data (CPD) is a promising instrument to assess the impact of purchases on health, but is limited by the need for manual scanning, a lack of access to data from multiple retailers, and limited information on product data and health outcomes. Here we describe the My Purchases cohort, a web-app enabled, prospective collection of CPD, covering several large retail chains in Denmark, that enables linkage to health outcomes. The cohort included 459 participants as of July 03, 2023. Up to eight years of CPD have been collected, with 2,225,010 products purchased, comprising 223,440 unique products. We matched 88.5% of all products by product name or item number to one generic food database and three product databases. Combined, the databases enable analysis of key exposures such as nutrients, ingredients, or additives. We found that increasing the number of retailers that provide CPD for each consumer improved the stability of individual CPD profiles and when we compared kilojoule information from generic and specific product matches, we found a median modified relative difference of 0.23. Combined with extensive product databases and health outcomes, CPD could provide the basis for extensive investigations of how what we buy affects our health.

## Introduction

Despite significant advancements in our understanding of human health, uncovering the lifestyle and environmental drivers of disease and aging will require new data resources in the years ahead^1^. While large contributors to lifestyle-related diseases and reduced lifespan, such as tobacco, alcohol, lack of exercise, and high-calorie diets have been identified, less is known about other exposures, such as less common food types, sanitary products and household chemicals^2^. Indeed, the causes of many diseases remain elusive, spurring a need to understand the combined effects of the many exposures we encounter throughout our lifetimes^3^. Despite increased focus on the commercial determinants of health, little is known about the impact of many consumer products on human health due to lack of high-quality, longitudinal studies analyzing product exposures and health outcomes^4^. Household purchases represent a significant proportion of these exposures. Research into consumer purchases, hereafter referred to as consumer purchase data (CPD), has increased dramatically in recent years. A recent review, overcoming a previously identified disparity in naming conventions^5^, concluded that “electronic sales data have the potential to transform the dietary assessment and worldwide understanding of dietary behavior” and that validation studies are warranted^6^. CPD has already been used to benefit patients directly by aiding outbreak investigations and harbors a large potential for further utilization provided residents and retailers consent to data collection^7,8^. Most often, researchers only have available CPD from one supermarket chain’s loyalty program or scanner data supplier and limited product information beyond item number and name are available for analysis^9^. Despite this, the CPD can be analyzed with a limited, but often highly informative purpose, such as providing realistic mixtures of chemicals for chemical risk evaluation^10^. To date, research interests have mainly been focused on diet, including validations against dietary surveys, aggregated use of time trends, and investigations into local food environments^6,11,12^.

**Figure 1:**
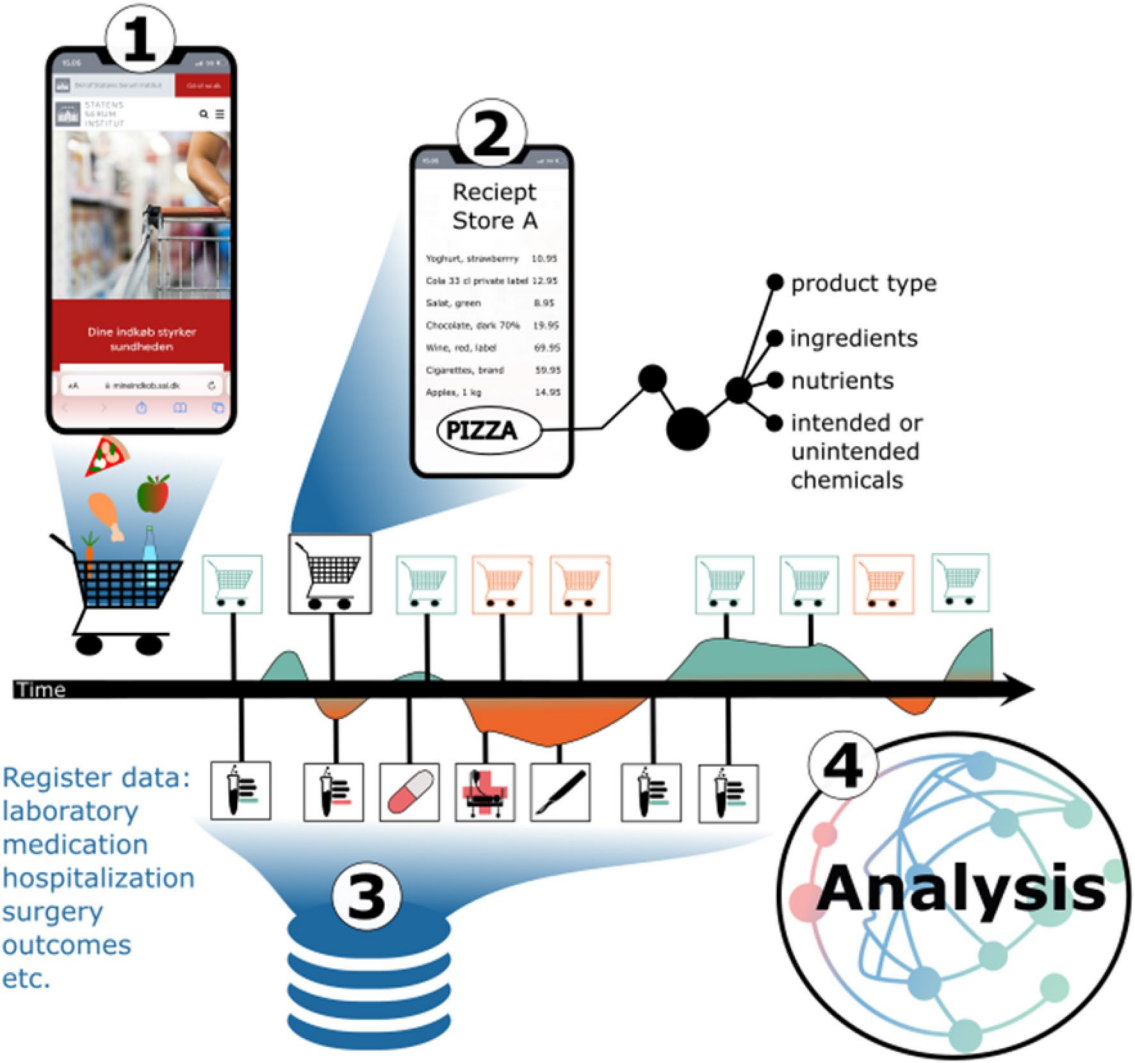
Illustrates the concept of the consumerome. (1) Recruitment of participants who consent to the continuous transfer of individual/household level CPD. (2) Establishment of near real-time pipelines, identification of the products sold, and enrichment of each product to enable broad assessment and identification of product type, ingredients, nutrients, intended and/or unintended chemicals etc. (3) Retrieval of individual-level information about health outcomes and trajectories. (4) Analysis of the impact of household consumer exposures on health.

Previous validation studies have focused mainly on data from major retailers and scanner data that require manual scanning by the consumer^9,11,13–15^. The validation studies have shown better alignment between purchase patterns of major nutrients in smaller households with a high degree of loyalty, and less alignment in larger households, especially in absolute values^11,16–18^. Whereas overall CPD patterns are stable, absolute consumption is very sensitive to the degree of loyalty/purchases made outside of retailers that the consumer data are collected from. There is a lack of studies analyzing data from multiple retailers to better understand the impact of retailer coverage and type on overall consumption patterns. Another potential limitation of CPD studies is the limited ability to sample a sufficiently large portion of purchases made and correctly match purchased products to relevant generic or specific product databases, moving from one type of exposure from one retailer to a broad assessment of many types of exposures from many retailers^19^. Such a hypothetical full coverage of all purchases made in a lifetime would approach a “consumerome”, mimicking the phenotypic variation of other “omics” as illustrated in Fig. 1.

As consumer data are collected from private companies, additional barriers to using CPD include concerns about data safety and security, personal privacy, data accuracy issues, and consumers not understanding the reason for data linkage or not using the services that produce these data^20^. Collection of data from loyalty programs often relies on manual scanning of loyalty cards and restrictions from retailers regarding use of data may potentially give way to issues with data continuity.

Here, using the My Purchases cohort as an example, we describe a comprehensive prospective collection of CPD, which is not dependent on a single retail chain, but rather a digital receipt solution gathering data from several retailers. We lay out potential possibilities to match products to generic or product specific databases and address the impact of having access to data from more than one retailer, before providing directions for future research.

## Methods

### Study design and population

The My Purchases cohort has had ongoing recruitment since June 2021. All Danish residents 18 years and older with a valid digital signature (MitID) and an account with the digital receipt solution can donate information about their purchases for research purposes. There are no specific selection criteria apart from the above. The consumer consents to transfer all previous digital receipts collected by the digital receipt solution and to transfer future receipts collected until they decide to leave the study or 2040, whichever comes first (the study page at https://mineindkob.ssi.dk/ has an option to leave the study).

### Data sources

#### Recruitment

Since June 2021, a GDPR-compliant, secure, encrypted web application developed by SSI, as part of the Human Exposome Assessment Platform (HEAP), has been in operation^21^. The solution allows consumers to provide legal consent to the automated retrieval of their past, current, and future CPD through a digital receipt solution, from a range of retailers (including three of the five largest retail chains in Denmark) as well as contact information and basic self-reported disease information at the time of consent^22^. Immediately following any purchase, the receipts are automatically generated and sent to the consumer’s account in the digital receipt solution app using the credit card information provided by the consumer to the digital receipt solution. The CPD is accessed once daily by Statens Serum Instutut through an application programming interface, providing a secure means of data transfer.

Integration with the Danish national digital signature solution ensures the valid consent and secure identification of participants, including retrieval of the Danish national Personal Identification Number (PIN)^23^. Using the PIN, the collected CPD can be linked to health outcomes from national registers or specific research projects, as detailed in the consent notice. Participants may choose to limit their consent to one research project or provide broader consent to all health-related research and may opt-in to be contacted again by the researchers.

Participants are also redirected to https://mineindkob.ssi.dk/ via a link in the digital receipt solution provider app, after a short introductory text in the data sharing setting of app, which includes a prompt to join the second time participants log-on. In addition, recruitment is supplemented by embedding links to the cohort landing page in questionnaires from two other studies^24,25^. Further targeted recruitment efforts using invitation letters are possible for additional projects.

#### Raw data collected from receipts

The CPD collected includes information found on receipts including price, discount, purchase date/time variables, and basic product information for all purchases made with debit/credit cards in partner retail chains. The basic product information includes a descriptive product item name e.g., “apples 6-pack” and the item number of the product. The item number corresponds to either a shorter supplier-generated barcode, or more often, the main world standard, the unique Global Trade Item Number (GTIN13) barcode. For a full list of all variables available, see Table S1.

#### Product databases

GS1 is a global standardization company that maintains large, country-specific, supplier-maintained product databases available through GS1 Trade Sync and has a wide range of product information, including item number, item names, ingredients, nutritional contents, storage information, allergens, and many other variables relevant to health^22^.

Kemiluppen is a product database with information on item number GTIN/barcode, product names and ingredients, and a three-level risk score. The risk score spans from A, where the product is free of a number of problematic substances, to B, where the product contains, for example, perfume that can cause allergies or substances that can harm the environment, to C, where the product contains potentially problematic ingredients e.g., hormone disruptors. Danish consumers can scan products in the application and receive information on the possible health effects of personal care products and cleaning products^23^.

Open Food Facts is a crowdsourced database of food products with ingredients, allergens, and nutritional facts. The database was started in 2012 and has been more extensively described elsewhere^26^.

Frida Food Database (hereafter referred to as Frida) is a generic food database covering more than 1000 different foods and has been described in detail elsewhere^27^.

Table S2 summarizes the main information found in the databases described above.

#### Matching specific product databases to purchased products

GTIN13 barcodes allow for further enrichment of products by matching limited product information from receipt data to additional product information from specific product databases. Current agreements allow us to match CPD with product information from the Global Standardization Organization GS1 (Trade Sync), allowing for comparison between different product sources^28^. Further, a collaboration with the Danish Consumer Council enables matching products from Kemiluppen^29^. Finally, we match data to the Open Food Facts database using the item number^30^.

#### Matching a generic product database to purchased products

Matching to generic product databases requires a preprocessing step that removes words and abbreviations from the product item name that could later interfere with partial word matching/regular expression (regex) matching steps. In addition, general product information that could be derived from the product name i.e., product weight, volume, origin, or type, e.g., organic, is identified and extracted for later use.

After preprocessing, each product item name is then matched to the generic product database. Here we used an adapted version of Frida^27^. The adapted Frida database includes the original Frida data plus columns of regular expressions representing brands, product type, specific and more generic descriptive words, taste/flavors, and fat content, which match to more than 1000 mutually exclusive Frida product types and some extra, mainly non-food categories that were not part of the original Frida database. The regex-matched database is designed to help select the product item names that best match the product type, using a scoring algorithm to flag unmatched products.

Since not all products are matched directly using the GTIN, misclassification can occur. Therefore, as a quality control measure, greedy matches, ambiguous stems (if any), and products not reaching thresholds (unmatched products) are repeatedly subjected to manual reviews and word stems are altered accordingly and iteratively to improve matching. In addition, for each product with information from more than one data source, e.g., GS1 and Frida, we compare nutrient information before finally performing a manual assessment of a random sample, as detailed in the statistics section.

### Statistics

In order to quantify the effectiveness of the applied regular expression matching of products and databases as well as to assess product variation between generic and specific product information, we compared nutrient information from all products that were matched to both Frida and GS1. The relative difference in nutrient information for each product and for each nutrient was estimated as the absolute difference between the two nutrient values obtained from Frida and GS1, respectively, divided by the maximum of the two values.

In addition, an analysis was performed to evaluate the accuracy in estimating an individual's purchase pattern when using a varying number of retailers. We focused on evaluating scenarios where we had data from only one or a subset of the retailers from each individual.

To do this, we calculated the percentage point difference in the proportion of purchases for the 20 most common food product groups (e.g., “bread,” “milk products,” “vegetables, raw,” “red meat,” “alcohol,” etc.) when using data from a single retailer compared to using information from all 34 retailers. In this analysis, positive values indicate items that are bought more frequently when considering data from one retailer compared to all 34, while negative values indicate items bought less frequently from one retailer. We then analyzed the variation of these differences across all individuals to evaluate how the accuracy of the estimated purchase pattern improved with an increasing number of retailers.

Finally, descriptive statistics were used to manually compare the proportion of products classified correctly according to unique products and total products sold. We excluded all products that were not matched to a product database or food database from the analysis.

R version 4.2.2 was used to conduct simple descriptive statistics^31^.

#### Ethics

Currently, many consumer loyalty programs mainly focus on using data to increase loyalty and revenue and consumers accept this in order to receive discounts or special offers. However, many consumers hesitate to share purchase data with researchers^20^.

Though fundamentally different in purpose with a focus on health, this study addresses the concerns of some consumers who worry about losing control of their data. As part of their consent, participants in the My Purchases cohort are given the option to limit data sharing and participation to only one study (see “Results” section).

The My Purchases cohort obtained consent from all participants and was approved by the Data Protection Agency officer at Statens Serum Institut (SSI) (journal no: 21/00949). The study is compliant with all guidelines and regulations, ethical, and IT-security requirements, and requires no further approval procedures under Danish law.

## Results

Between June 22, 2021 and July 03, 2023, a total of 459 participants were recruited. Of these, 426 (92.8%) consented to all registered studies and of those, 282 (61.4%) also approved participation in all future studies. 64 (13.9%) did not consent to the sharing of data in an identifiable form beyond SSI. No participants consented to participation in less than two studies. 12 (2.6%) participants had one or no receipts registered in the data and were excluded. General information regarding the number of participants, products bought, total number of receipts, and self-reported disease are shown in the flowchart in Fig. S1.

### Characteristics of participants

Table 1 shows basic demographics, such as age and sex as well as retailer “loyalty” ranging from shopping in a single retailer to attending a range of retailers. Shopping frequency varied greatly with an interquartile range (IQR) of 5.4–12.6 and an average of 8.4 receipts per month, with receipts being available from about half of the cohort for the past 5 years or more. The median total expenditure of each participant per month was roughly 1900 Danish Kroner (253 Euros [per March 30, 2023]).

**Table 1:** Age, sex, purchase behavior, and follow-up distribution for the 447 cohort members.

Characteristics and purchase behavior	N = 447
Age^a^	49 [37–59] (19–86)
Sex	
Male	198 (44%)
Female	249 (56%)
No. products^a^	3,629 [1640–6846] (9–30,303)
Average product price (dkk)^a^	21.7 [18.3–26.5] (9.0–516.9)
No. receipts^a^	370 [168–654] (2–1675)
Receipts pr. month^a^	8.4 [5.4–12.6] (1.1–47.1)
Money spent pr. month (dkk)^a^	1863 [1238–3073] (158–23,194)
Enrollment time^a^	
< 1 Year	36 (8.1%)
1–2 Years	51 (11%)
2–3 Years	32 (7.2%)
3–4 Years	36 (8.1%)
4–5 Years	36 (8.1%)
+ 5 Years	256 (57%)
Inactive time (months)	8 [0–24] (0–76)
No. merchants used^a,b^	13 [10–17] (1–27)

^a^Reports median [IQR] (min–max).

^b^Defined as whole months with zero purchases.

### Receipt coverage over time

The digital receipts solution has been present in the Danish market since 2014 and has increased the number of unique retailers included in the provider from 15 in 2015 to 34 in 2022. In the My Purchases cohort, data from 140 participants was available going as far back as 2015 gradually increasing to the presented number of participants. Throughout the study period, the annual number of receipts (5952–40,440), products (11,299–42,681), and total products bought (79,586–447,186) increased, reflecting the rise in the number of participants generating receipts, as well as wider coverage of retailers and the introduction of new products to market. Out of the 223,440 unique products purchased, the distribution of products purchased was skewed towards a few frequently purchased products; thus the 1000 most frequently purchased products accounted for 38.8% of all products purchased (Fig. S2).

### Product enrichment

After collection, all data undergoes an enhancement step where each product was matched to specific or generic information coming from different data sources including GS1, Frida, Open Food Facts, and Kemiluppen, which combined, provide information regarding product type, ingredients, nutrients, intended chemicals, and more. GS1, Open Food Facts, and Kemiluppen are matched using GTIN number, whereas Frida uses word-recognition to match the product name.

**Figure 2:**
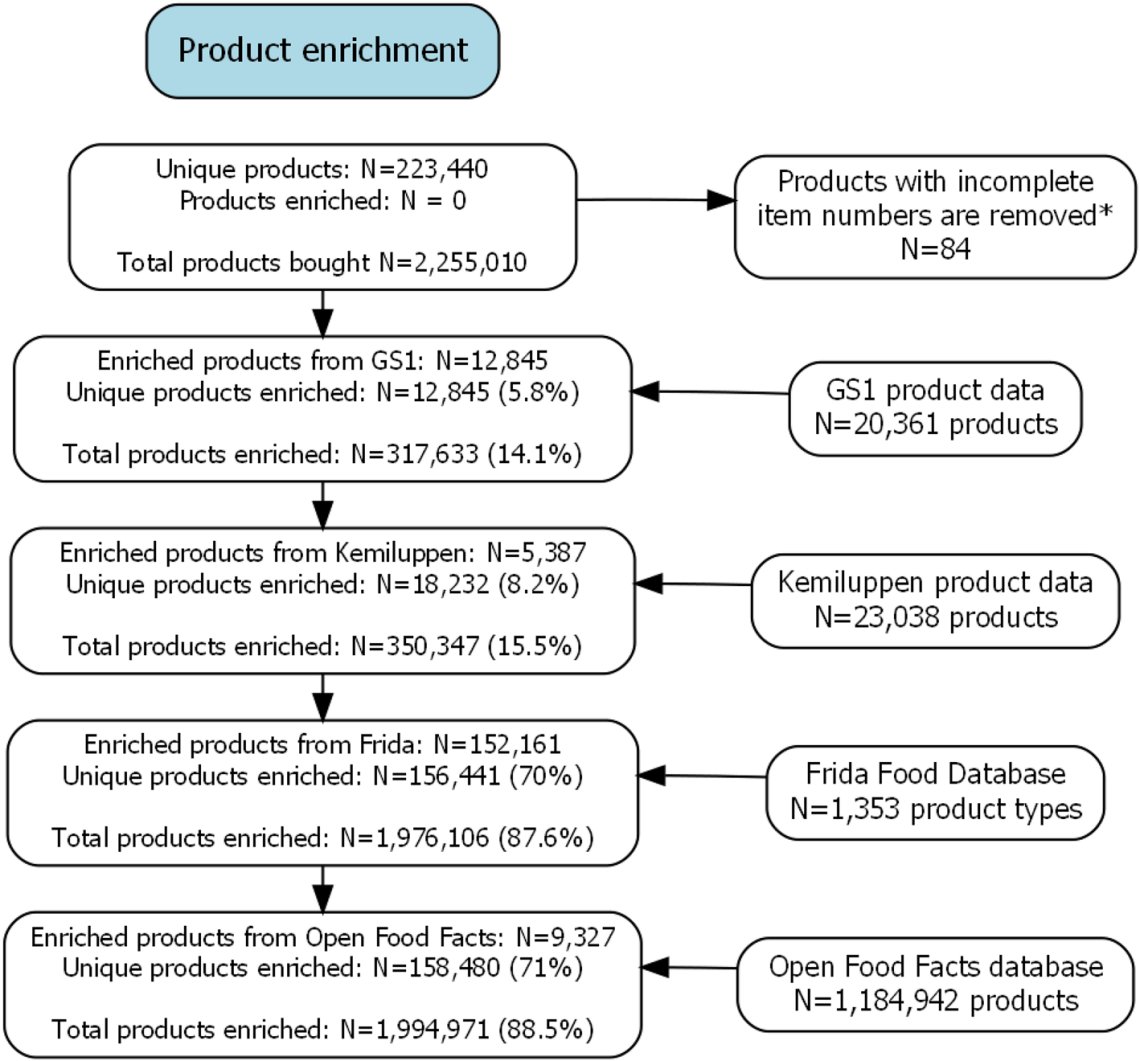
Detailed flowchart of the enrichment of product information. *Excluded products with item-numbers that had either more than 14 digits or less than 3 digits.

The entire product enrichment pipeline is outlined in Fig. 2 and enables the retrieval of information beyond the product item name and item number for 71% of all unique products and 88.5% of the total amount of products purchased. Of these, 18,232 products were directly matched to specific products from either GS1 or Kemiluppen using the GTIN code.

#### Differences in nutrient values between generic and specific databases

**Figure 3:**
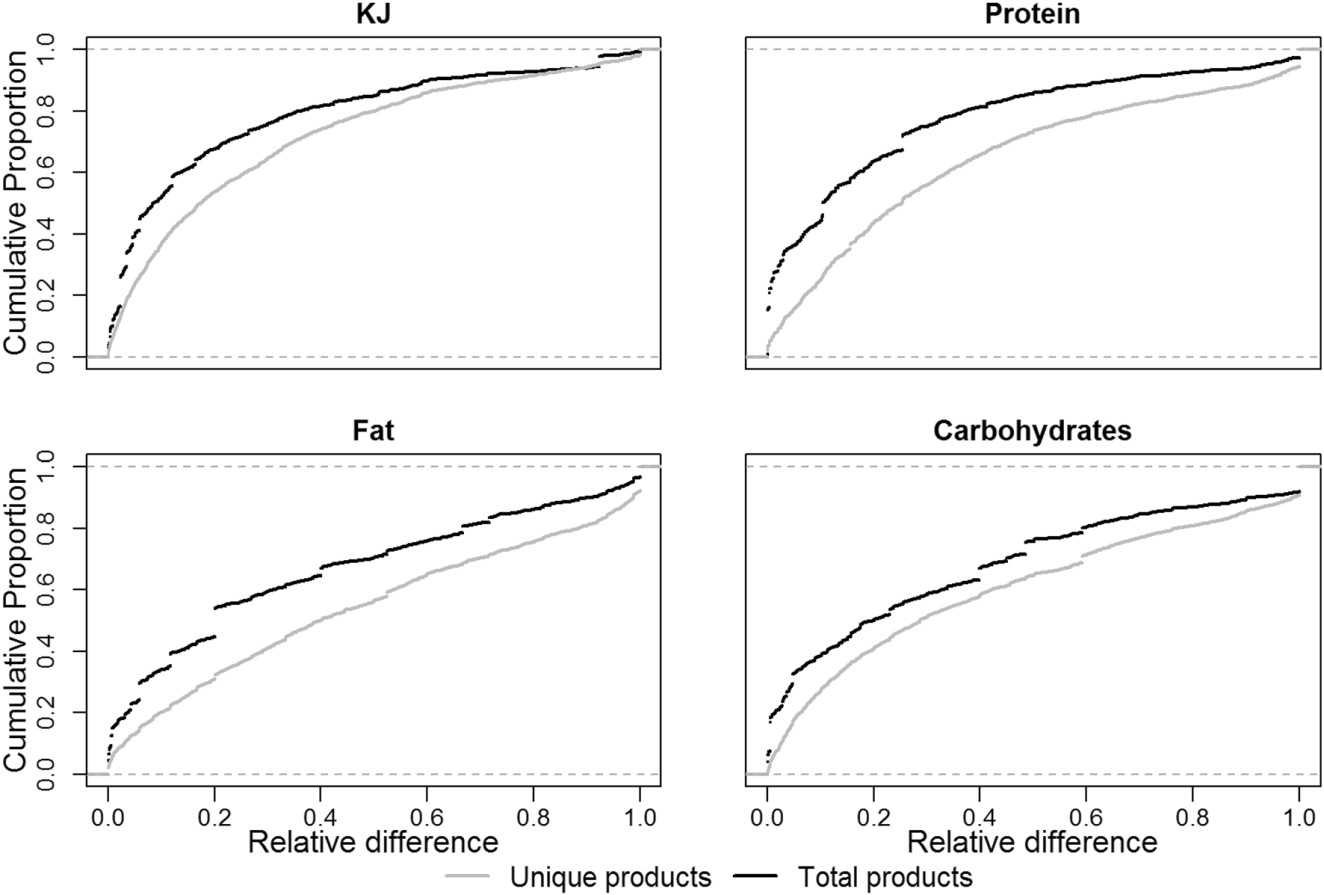
Comparison of nutrient information from Frida and GS1 using a cumulative distribution of relative difference of product information concerning KJ, protein, fat, and carbohydrates.

Figure 3 shows the cumulative distribution of the relative difference for all unique products (and total products) for kilojoule (KJ), fat, protein, and carbohydrate content. Table S3 shows the distribution of the relative difference in KJ for all unique products found both in the branded GS1 database and the generic Frida database. The median modified relative difference was 0.23, but the median varied greatly between product groups.

#### Differences in proportion of purchases by number of included retailers

**Figure 4:**
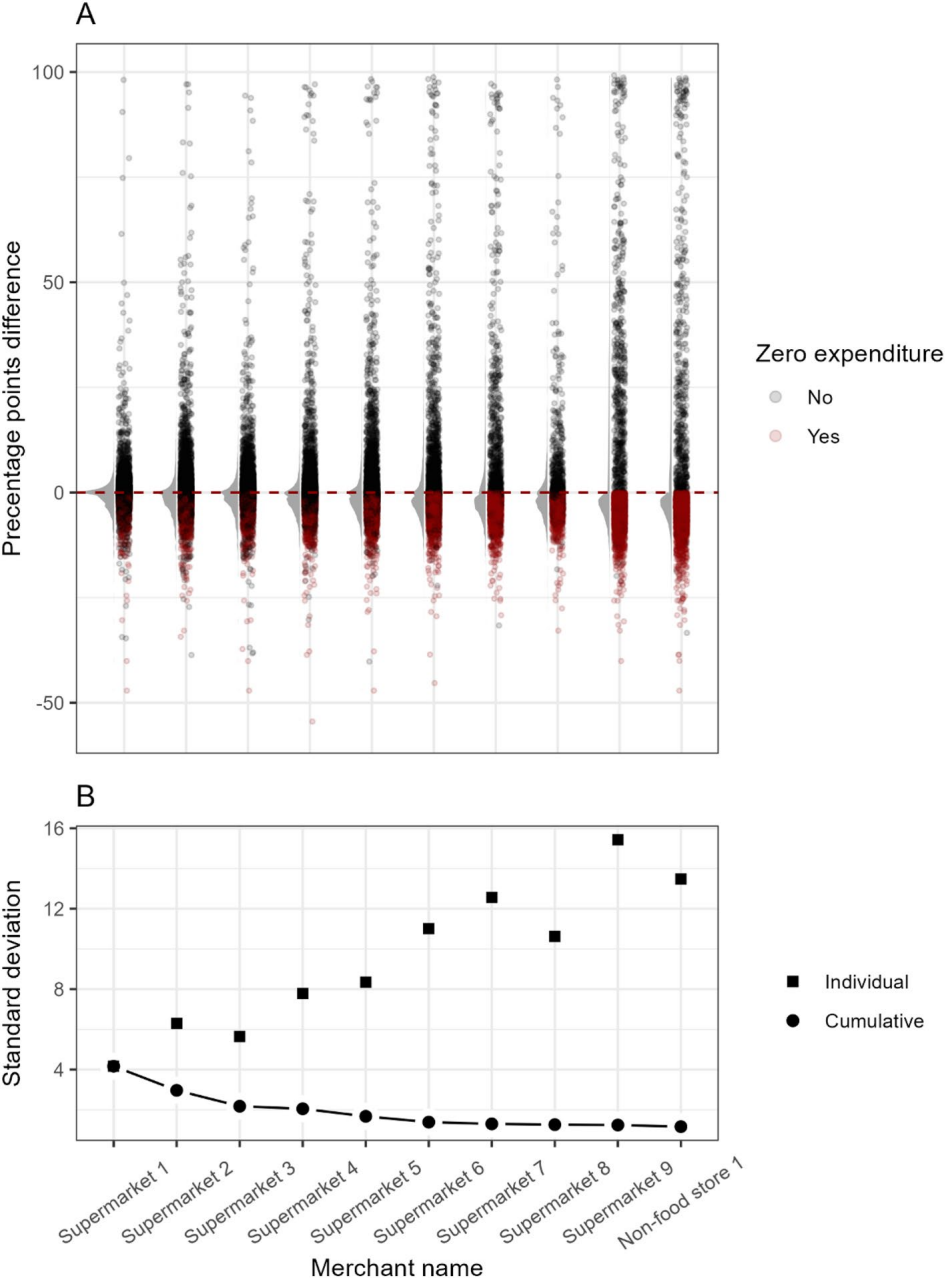
Change in the estimated proportion of purchases across the 20 most frequent food related product groups for all participants using only information from a single retailer compared to using the information from all 34 retailers. Results are shown for the 10 largest retailers and are ranked based on total purchases. (**A**) Each point corresponds to a single difference in the fraction of purchases for one food related product for a random participant. Positive difference denotes a higher purchase fraction than expected. Red dots indicate that the participant had zero registered purchases for a particular retailer, when only a portion of data was used. (**B**) Standard deviation of the estimated purchase differences when the retailers are used individually (Square) and cumulatively combined (Circle).

Figure 4 illustrates the importance of having access to data from multiple retail chains. Panel A shows the change in each individuals estimated proportion of purchases across the 20 most frequent food-related product groups when using only information from one retailer compared to using all 34 retailers. Panel B shows the variation for the same change in the estimated proportion of purchases and how including more retailers reduces variation approaching the pattern found when using all 34 retailers. Table S4 shows the median (10th; 90th percentiles) relative difference in the estimated proportion of purchases from each product group when using only information from 1, 2 or 4 of the largest retailers compared to using information from all 34 retailers. For the most frequently bought product categories, the median change was − 8.6% (− 100%; 83.7%) when using data from only one retailer and 1% (− 16.7%; 14.8%) when using data from four retailers. For less frequently bought categories such as tobacco, dental products, and cleaning products, the median changes across all product groups when using data from one retailer versus four retailers were − 13.1% (− 100%; 91.2%) and 1% (− 21.1%; 16.3%), respectively.

#### Manual assessment of product matching to generic product database

We also extracted 1000 random products and assessed the product matching to Frida manually. A total of 74.8% of unique products and 89.2% of all products purchased by volume were exact matches e.g., “skimmed milk” and “skimmed milk”, whereas 77.8% of unique products and 92.5% of all products purchased by volume were group matches e.g., matching milk correctly but not the subtype of milk product.

### Self-reported diseases and conditions

In total, 202 (44%) participants reported at least one disease or condition, with 40 (8.7%) reporting two, 25 (5.5%) reporting three, and 31 (6.8%) reporting four or more. Diseases and conditions most frequently reported were back pain (n = 33), hypertension (n = 22), type 2 diabetes (n = 21), irritable bowel disease (n = 20), allergy (any) (n = 20), and Crohn's disease (n = 16). Conditions reported by more than five participants are shown in Table S5.

## Discussion

Here, we describe one of the most comprehensive, prospective collections of consumer purchase data (CPD) to date. 459 participants have registered and additional recruitment is active and ongoing, enabling continuous prospective collection of CPD. Though this number may seem small, we have collected CPD spanning up to 8 years of purchases, currently from 34 retailers, including several large supermarket chains as well as one generic food database and three specific product databases. The various data sources enable broad exposure assessment of otherwise hard to obtain information on consumption of grocery products including, food, tobacco and alcohol, sanitary products, and chemicals.

We found that comprehensive enrichment, which both quantifies known product groups contributing to disease, such as smoking and alcohol consumption and allows for broad exposure assessment of otherwise hard-to-access exposures, such as indoor pollutants (e.g., candles and sprays) and chemicals in cosmetics is possible^10^. Although we report on self-reported diseases, the continuous CPD collection can be linked to health outcomes, enabling register-based, longitudinal analysis of how lifestyle related factors are associated with disease onset, propagation, or cessation.

All 223,440 unique products sold to participants could, in principle, cause disease, as is often the case in foodborne outbreaks^7^. Apart from the most commonly purchased products and products with high brand recognition, it may be challenging to identify such products using questionnaires due to limited recall by participants. If other diseases or changes in disease trajectory are triggered by the purchase of a single product, a raw CPD sample with high coverage could thus be superior to questionnaires, as was the case in a recent simulation study^8^. Such studies are however highly dependent on participants making traceable purchases and the retailers providing data^16^. We found a high dispersion in the proportion of purchases from each product group when data from only one retailer was used, with a reduced dispersion as data from more retailers were combined. All product groups, including important groups such as tobacco, had a relative fractional difference of 100 in the 10th percentile when using data from only one retailer. This could result in the possible misclassification of purchase behavior, such as classifying an individual as an apparent non-smoker, when in reality, the individual is buying cigarettes at another retail chain. Our results illustrate that having data from only one retailer not only increases the risk of measurement error, but also that this measurement error was dramatically reduced as the number retailers that contribute data increased. This is in line with findings from studies using loyalty programs, where a higher degree of loyalty was associated with better agreement between consumer data profiles and food frequency questionnaires^16^. This is an important finding for future studies comparing consumption patterns to data from e.g., food frequency questionnaires and for studies of consumer purchase data and health.

Another factor affecting our ability to determine the impact of multiple products on health is that we are able to collect a broad range of information beyond the product name and number. Here, information was retrieved beyond the product item level for 89.2% of all products sold, enabling analysis beyond the product item name or item number and allowing for the assessment of ingredients, nutrient information, and many other exposures relevant to health^11,13,19^. As products are matched to Frida and a range of custom-made, non-food categories using regular expressions, some mismatches are expected. Moreover, product-specific discrepancies, such as in caloric content within the same product group due to differences between average product values in the food database and the specific brand (e.g., different spread products) are also expected. We quantified differences between the average product values of generic products in Frida and the GTIN specific product information from GS1. Though direct comparison is not possible due methodical differences, we found the median modified relative difference for KJ to be 0.23, which appear higher than what was previously shown in a large British study where products were manually compared^32^. These findings are only partially explained by generic versus branded product differences and some variation is due to misclassification of products e.g., low versus full-fat products or residual errors in the matching algorithm. Manual assessment of a sample of 1000 randomly selected matched products revealed that 75% of unique products and 89% of all products purchased by volume were good matches to generic products. Additionally, 78% of unique products and 92% of all products purchased by volume matched on the group level. This highlights the need for continuous improvement of the matching algorithm, as the current framework allows for and encourages further development, as well as the use of other approaches including natural language processing. Despite these challenges, overall consumption patterns for top product groups found in Table S3 corresponded well to those published by Statistics Denmark^33^. More promising is direct matching to structured specific product databases such as GS1 Trade Sync, Kemiluppen, and Open Food Facts^28–30^. The unique GTIN13 maintained by GS1 enables direct matches, but the coverage of these databases may vary by country and for GS1, the coverage is producer-dependent, where some product producers do not allow research-related access. For the foreseeable future, efforts should focus on combining specific and generic product databases, where supplier generated product information and information from specific product databases should be preferred over generic information.

In this study, we combine name matching and GTIN matching to create a near real-time enrichment pipeline for purchased products. This framework allows us to follow key health determinants, such as tobacco, alcohol, dental products, and diet over time, and thereby investigate time-varying exposures, possible associations with health outcomes, and resultant targets of interventions^24,34,35^. Though the outcomes described here were self-reported, the consent given by participants allows researchers to further enrich the cohort with information from Danish registers. The Danish registers provide access to outcomes such as prescription data, microbial and biochemical laboratory results (calprotectin, C-reactive protein, and cholesterol), outpatient and hospital visits, and surgical procedures, in addition to a number of key health and economic outcomes as well as spatial and social data. These registers have been extensively described elsewhere^36–39^. In addition to register data, other research projects may add further data to the cohort, enabling the future assessment of onset, flare-ups, alleviation, and cessation of a wide range of diseases^24,25^.

### Limitations

This study has a number of limitations. First, the age and sex distributions of the cohort differ from the Danish population as a whole, with the cohort being older and predominantly female, highlighting the risk of selection bias. However, the potential for analytically managing this bias is promising due to the large amount of longitudinal data for both the cohort and the total population^14^. Other challenges include purchases being made at other retailers, loss to e.g., food waste, the level of detail in product grouping, and the knowledge gaps regarding what is being consumed and by whom (e.g., eating out, at a friend’s, and within a household)^14,40,41^. Though identifying individual consumption is a challenge, Danish registers allow for identification of household information, including single households where this is less of an issue^14^. Furthermore, our database does not include all retailers and loss of data due to delays in the participant's updating of the digital receipt app when credit/debit cards expired and/or are renewed is a challenge and the most likely cause of the average 7 months without purchases found in this cohort. This is also reflected in the lower-than-expected total expenditure of 1863 Danish Kroner per participant per month, compared to the mean expenditure of 3164 for each Dane published in 2019 by Statistics Denmark^42^. In addition to the lack of complete consumerome coverage, finding available product data beyond what is reported via receipts is also a challenge. Among purchases made by participants, 24% of unique products and 11% of all products purchased by volume were not uniquely matched. Further, though a larger amount of information is available for generic products, many other key variables, such as weight or volume, are only available to a limited extent from product names and need structured product specific databases such as GS1 to be assessed^14,40^.

### Strengths

Major strengths of the current cohort are the broad number of retailers provided in the sample, the ability to ascertain the impact of having CPD from more than one retailer, the length of follow up (up to 8 years of CPD), the ability to enrich and classify the majority of products that combined, enable the ability to investigate multiple, hard-to-assess exposures over prolonged time frames with minimal time or effort required from the participants and no social desirability bias or recall bias. Many of the technical limitations mentioned above can be addressed by further improving the product enrichment pipeline, including adding Natural Language Processing approaches, providing services that encourage individuals to provide CPD for the entire household, or by adjusting the data for household composition that could work in tandem with targeted questionnaires to address identified knowledge gaps^14,40^.

Another strength is the ability to use the personal identification number provided by participants to collect key covariates from Danish registries, including household size, income, education, and social data, in addition to other exposure data and health outcomes, as detailed above.

### Implications

The My Purchases cohort combines consumer purchase information with health outcomes. To ensure large-scale collection of CPD, creating services that provide insights to participants, while addressing the need for information, choice, and appropriate safeguards is evident^43^. Options such as being able to select/deselect various categories of research and selecting different transaction data/CPD data sources could improve participants trust in the recipient organizations^44^. In the future, CPD could enable post-marketing, epidemiological assessment of products and help unveil the commercial determinants of health, including health effects of additives and foods introduced to consumers^4^. Such information may then inform politicians and key institutions, such as European Food Safety Authority (EFSA) and European Chemicals Agency (ECHA) of such effects^45,46^ and enable novel approaches to changing consumer purchase behavior using incentives or apps targeting consumer choices^34,47^. With time and in combination with other sources, the exposures to biological pathways at different life stages and identification of early signs of health damage caused by environmental factors could be discovered and real-time lifestyle advice ameliorating or preventing the impact of these factors could be directly communicated to consumers.

### Conclusion and directions for future research

Increasing the number of retailers that provide CPD improves the stability of assessment of individual CPD profiles. Combined with extensive product databases, this could provide a broad assessment of individual exposures from commercial activities approaching a “consumerome”, that could in time provide the basis for investigations of how what we buy affects our health. Future studies enabling analysis of the impact of the consumerome on health should:

1. Establish large cohort(s) with participants that consent to the continuous transfer of individual/household level CPD and retrieve person-level information about health outcomes and trajectories as well as enable the analysis of the impact household consumer exposures may have on health.
2. Further develop near real-time pipelines, identify the products sold, and enrich each product to enable broad assessment and identification of product type, ingredients, nutrients, intended or unintended chemicals, and standardized reporting of key metrics concerning product matching efficiency, completeness of data, and retailer coverage.
3. Develop analytic methods utilizing the multiple time-varying exposures, taking into account the complex longitudinal data structure, including repeated measurements and missing data.

Thus, additional research and funding are needed to further explore the potential of the consumerome to uncover novel lifestyle disease associations and to translate potential targets of prevention into safer products and actionable lifestyle advice. The authors call on key retailers across the EU (and worldwide) to support research access to CPD, as the number and thus coverage of retailers in the CPD samples are crucial to assess consumer exposures accurately.

### Supplementary Information

Supplementary Information.

## Data Availability

Data on participants are not publicly available but agreements regarding the transfer of code can be made by contacting the author. A framework enabling data access is underway, either via Statistics Denmark or a similar, secure platform, as is planned by HEAP^48^. The product enrichment pipeline matching products to generic Frida product categories is available at https://github.com/ThorJunkerSSI/ConsumerData. A simulated dataset (that does not reproduce the main results, but allows other researchers to run the code) is also available on https://github.com/ThorJunkerSSI/ConsumerData. The raw data is currently not publicly available in order to secure the confidentiality of retailers and participants according to their consent. Data from Frida and Open Food Facts are available from their respective webpages at https://frida.fooddata.dk/data?lang=en and https://world.pro.openfoodfacts.org/cgi/import_file_upload.pl. Kemiluppen data is available by specific request https://taenk.dk/medlemskab/kontakt-medlemsservice request and GS1 share their product data through an API, where Keys are obtained through membership of the GS1 Trade Sync system, which is available for an annual fee https://www.gs1.dk/services/gs1trade-sync.
