# Supplementary material for: Assessing household lifestyle exposures from consumer purchases, the My Purchases cohort
Source: Sci Rep. 2023 Dec 7;13:21601. doi: 10.1038/s41598-023-47534-6 (PMC10703931; doi:10.1038/s41598-023-47534-6)
Supplement: Supplementary file 1 — Supplementary Information. [file 41598_2023_47534_MOESM1_ESM.docx]

**Assessing household lifestyle exposures from consumer purchases, The My Purchases cohort**

**Authors**

Frederik T. Møller*, Thor Grønborg Junker, Kathrine Kold Sørensen, Caroline Eves, Jan Wohlfahrt, Joakim Dillner, Christian Tobias Torp-Pedersen, Bartlomiej Wilkowski, Steven Chong, Tune H. Pers, Victor Yakimov, Heimo Müller, Steen Ethelberg, Mads Melbye

**Supplementary materials**

**Table S1 Consumer Purchase data variables, descriptions, and example values**

| **Name** | **Description** | **Example values** |
| --- | --- | --- |
| **Receipt ID** | Receipt ID | Receipt_3773 |
| **Item name** | Description of product from receipt | Banana 4pcs |
| **Item number** | Itemnumber or GTIN13/EAN/UPC | 5710815000014 |
| **Amount** | Number of products purchased | 1 |
| **Item price** | Price in dkk | 12.95 |
| **Rabat** | Discount in dkk | -1.00 |
| **Purchdate** | Date of purchase | 2021-05-02 |
| **Week no** | Week of purchase | 5 |
| **Monthno** | Month of purchase | 2 |
| **Yearno** | Year of purchase | 2021 |
| **Merchant name** | Name of retailer | supermarket name |

**Table S2**

Generic and specific product databases

| Database | Match | Variables | Product/group examples | Data access |
| --- | --- | --- | --- | --- |
| Frida | Name match | Macronutrients, Micronutrients | Tomato, raw  Soft drink, no sugar added  Pizza, frozen | <https://frida.fooddata.dk/data> |
| Kemiluppen | GTIN/UPS/EAN/itemnumber | Ingredient list, Classification | Product name: Toothpaste  Category: Soap and Hygiene  Ingredients: Aqua, hydrated silica …  Classification: B | <https://taenk.dk/kemi/plejeprodukter-og-kosmetik/kemiluppen-tjek-din-personlige-pleje-uoensket-kemi> |
| Openfoodfacts | GTIN/UPS/EAN/itemnumber | Quantity, Packaging, Categories, Ingredients, Macronutrients | Product: Nutella  Quantity: 350 g  Categories: Breakfast, Spreadable products  Nutri-Score: E  Nutritional Information | <https://world.openfoodfacts.org/data> |
| GS1 | GTIN/UPS/EAN/itemnumber | Ingredients, Macronutrients | Segment 50000000 - Food/Beverage/ Tobacco  Family 50200000 – Beverages  Class 50202600 - Coffee/Coffee Substitutes  Brick 10008178 - Coffee - Whole Beans | <https://gpc-browser.gs1.org/> |

**Table S3.**

Summary table of the modified relative error difference in KJ between Frida and GS1

|  | Quantile | | | | |  |  |
| --- | --- | --- | --- | --- | --- | --- | --- |
| Product group | Min | 25% | 50% | 75% | Max | Products in GS1 | N |
| **Mean** | **0.00** | **0.10** | **0.23** | **0.41** | **0.89** | **8,949** | **1,391,223** |
| Fruit, raw | 0.00 | 0.35 | 0.71 | 0.86 | 0.99 | 157 | 187,733 |
| Vegetables, raw | 0.00 | 0.31 | 0.57 | 0.81 | 1.00 | 161 | 173,003 |
| Milk products | 0.00 | 0.03 | 0.19 | 0.42 | 0.94 | 969 | 128,107 |
| Confecture, chocolate, ice cream, and sugar | 0.00 | 0.03 | 0.10 | 0.33 | 1.00 | 1,418 | 117,474 |
| Bread | 0.00 | 0.06 | 0.14 | 0.25 | 0.56 | 669 | 87,599 |
| Sugary drinks | 0.00 | 0.09 | 0.28 | 0.79 | 1.00 | 438 | 70,875 |
| Non-sugary drinks | 0.00 | 0.49 | 0.92 | 0.98 | 1.00 | 490 | 70,409 |
| Butter, oil and dressings | 0.00 | 0.05 | 0.12 | 0.31 | 0.88 | 398 | 62,830 |
| Processed red meat | 0.00 | 0.06 | 0.18 | 0.36 | 1.00 | 434 | 62,411 |
| Vegetable products | 0.01 | 0.20 | 0.39 | 0.63 | 0.94 | 145 | 56,403 |
| Poultry | 0.00 | 0.26 | 0.53 | 0.60 | 0.84 | 156 | 49,644 |
| Cookies, crackers, biscuits and cakes | 0.00 | 0.03 | 0.07 | 0.12 | 0.71 | 701 | 47,840 |
| Cheese | 0.00 | 0.12 | 0.21 | 0.33 | 0.83 | 803 | 45,664 |
| Red meat | 0.00 | 0.31 | 0.50 | 0.59 | 0.81 | 141 | 42,616 |
| Fish and other aquatic animals | 0.00 | 0.11 | 0.20 | 0.39 | 0.86 | 231 | 39,624 |
| Alcoholic drinks | 0.00 | 0.21 | 0.42 | 0.83 | 1.00 | 294 | 35,580 |
| Ready meals | 0.00 | 0.10 | 0.20 | 0.34 | 1.00 | 260 | 34,307 |
| Salty snacks | 0.00 | 0.01 | 0.04 | 0.08 | 0.87 | 195 | 28,362 |
| Fruit products | 0.00 | 0.10 | 0.21 | 0.40 | 0.94 | 70 | 27,012 |
| Rice and pasta | 0.01 | 0.03 | 0.27 | 0.61 | 0.98 | 144 | 23,730 |

**Table S4.**

**Median (10th; 90th percentiles) of the relative change in the estimated proportion of purchases by product group for all participants using only information from 1, 2, or 4 retailers compared to using all 34 retailers**

|  | **Number of retailers** | | |  |
| --- | --- | --- | --- | --- |
| **Products Group** | **One** | **Two** | **Four** | **N products** |
| Fruit, raw | -3.4 (-100; 56.5) | 1.5 (-43.1; 37.7) | 2.2 (-6.5; 15.4) | 187,733 |
| Vegetables, raw | -3 (-100; 44.3) | 1.3 (-39.2; 30.2) | 1.7 (-5; 13.1) | 173,003 |
| Milk products | -6.4 (-100; 48.1) | 0.9 (-53.9; 35) | 1.8 (-7.7; 12.3) | 128,107 |
| Confecture, chocolate, ice cream, and sugar | -4.1 (-70.5; 98.9) | -2.9 (-39.3; 55.1) | -2.1 (-19.7; 7.4) | 117,474 |
| Bread | -6.8 (-100; 71.9) | -1.3 (-55.4; 45.2) | 0.5 (-18.2; 11.7) | 87,599 |
| Sugary drinks | -10.8 (-100; 105.3) | -1.9 (-62.5; 64.8) | 0.2 (-19.6; 16.9) | 70,875 |
| Non-sugary drinks | -9.8 (-100; 124.7) | -4.8 (-62.7; 66.8) | -1.8 (-27.7; 10) | 70,409 |
| Butter, oil and dressings | -6 (-100; 69.8) | -0.7 (-52.9; 44.1) | 0.9 (-11.7; 12) | 62,830 |
| Processed red meat | -4.3 (-100; 64.3) | 2.5 (-61; 45.6) | 3.6 (-3; 18.8) | 62,411 |
| Vegetable products | -8.7 (-100; 65.9) | 0.6 (-59.5; 44.8) | 1.5 (-12.4; 16.6) | 56,403 |
| Poultry | -5.7 (-100; 82.6) | 0.9 (-61.4; 53.9) | 1.9 (-8.3; 16.2) | 49,644 |
| Cookies, crackers, biscuits and cakes | -10.9 (-100; 124.5) | -2.8 (-64.3; 72) | -0.5 (-22.5; 14.7) | 47,840 |
| Cheese | -7.2 (-100; 66.3) | 0.6 (-60.6; 44.1) | 1.6 (-9.9; 14.5) | 45,664 |
| Red meat | -9.8 (-100; 78.3) | -1.2 (-60.8; 48.9) | 0.7 (-15.7; 12.2) | 42,616 |
| Fish and other aquatic animals | -12 (-100; 90.2) | -0.1 (-77.1; 61.5) | 1.5 (-13.8; 20.9) | 39,624 |
| Alcoholic drinks | -23.3 (-100; 117.8) | -10.6 (-77.7; 70.1) | -4.1 (-43.9; 7.1) | 35,580 |
| Ready meals | -10.5 (-100; 95.9) | -2 (-72; 61.9) | 0.9 (-15.9; 15.5) | 34,307 |
| Salty snacks | -15.7 (-100; 105.6) | -5.1 (-79.8; 71.8) | -1.9 (-29.9; 12.2) | 28,362 |
| Fruit products | -18.3 (-100; 105.3) | -0.4 (-100; 71.7) | 1.4 (-14.5; 19) | 27,012 |
| Rice and pasta | -15.3 (-100; 98.4) | 1.5 (-100; 66.6) | 2.9 (-7.6; 21.1) | 23,730 |
| **Total of most frequent groups** | **-8.6 (-100; 83.7)** | **-0.7 (-63; 53.1)** | **0.9 (-16.7; 14.8)** | **1,391,223** |
| Tobacco | -100 (-100; 172.9) | -8 (-100; 124.4) | 2.6 (-30.1; 19.3) | 13,659 |
| Soap and Hygiene | -60.1 (-100; 145.4) | -17.4 (-100; 99.5) | -2.6 (-48.1; 16.3) | 10,030 |
| Dental products | -54.7 (-100; 137.2) | -22.2 (-100; 90.7) | -4.6 (-53.3; 11) | 9,127 |
| Cleaning products | -70.3 (-100; 137) | -16.7 (-100; 92.4) | 0.1 (-45.5; 20.2) | 4,034 |
| **Total** | **-13.1 (-100; 91.2)** | **-1.6 (-87.2; 60.2)** | **1 (-21.2; 16.3)** | **1,501,838** |

**Table S5**

The most frequently occurring self-reported diseases and conditions

| Disease | n |
| --- | --- |
| Back-pain | 33 |
| Hypertension | 22 |
| Type 2 diabetes | 21 |
| Allergy | 20 |
| Irritable bowel disease | 20 |
| Covid-19 | 18 |
| Crohn’s Disease | 16 |
| Astma | 14 |
| Psoriasis | 13 |
| Depression | 10 |
| Ulcerative Colitis | 10 |
| ADHD | 9 |
| Migraine | 8 |
| Fibromyalgia | 7 |
| High cholesterol | 7 |
| Arthrosis | 6 |
| COPD | 6 |
| PTSD | 6 |
| Sarcoidosis | 6 |
| Coeliac disease | 5 |
| Sclerosis | 5 |

**Figure S1:**

Figure S1: Flowchart of recruitment and available data


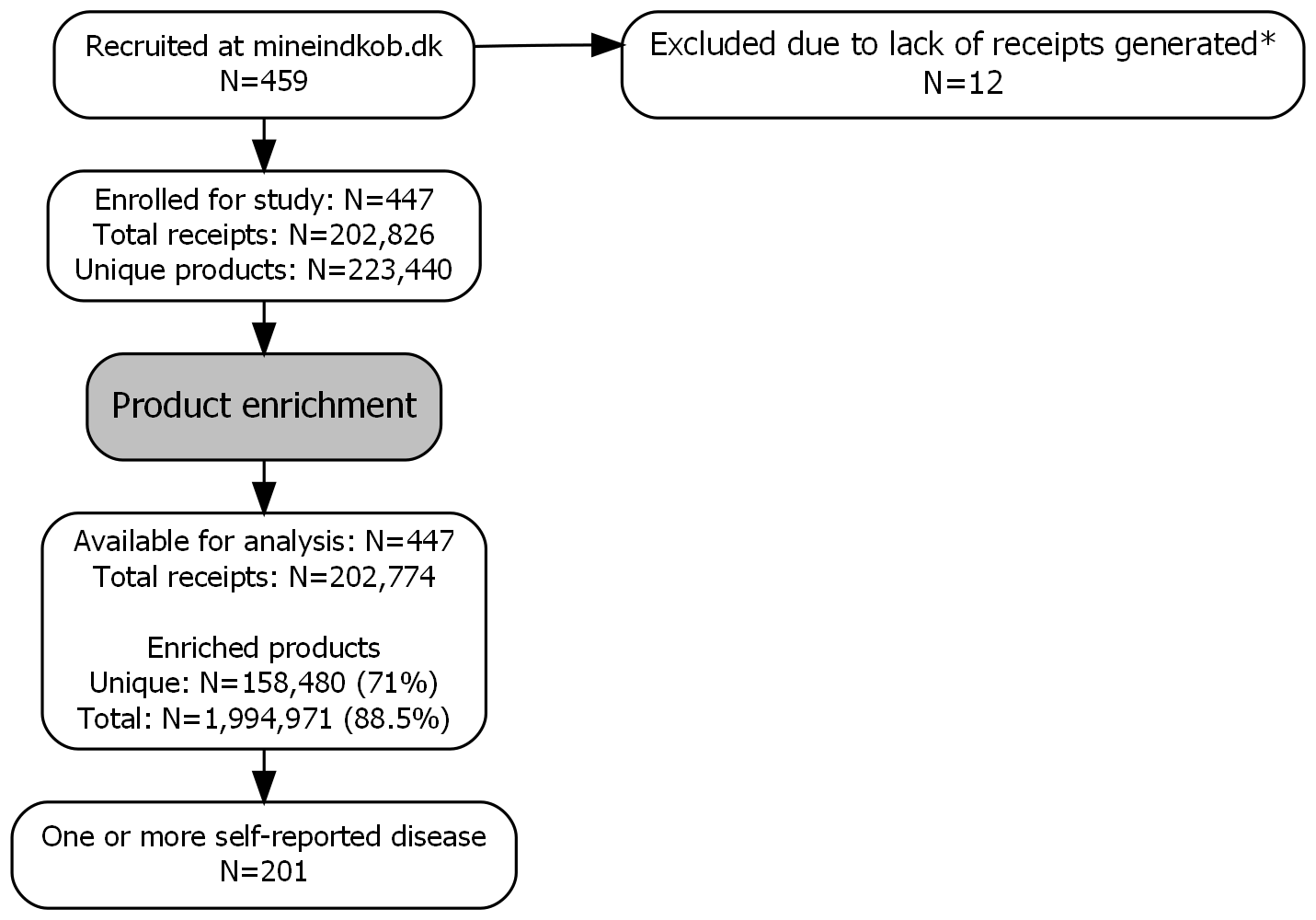


*Participants with 1 or no receipts registered were removed.

**Figure S2**


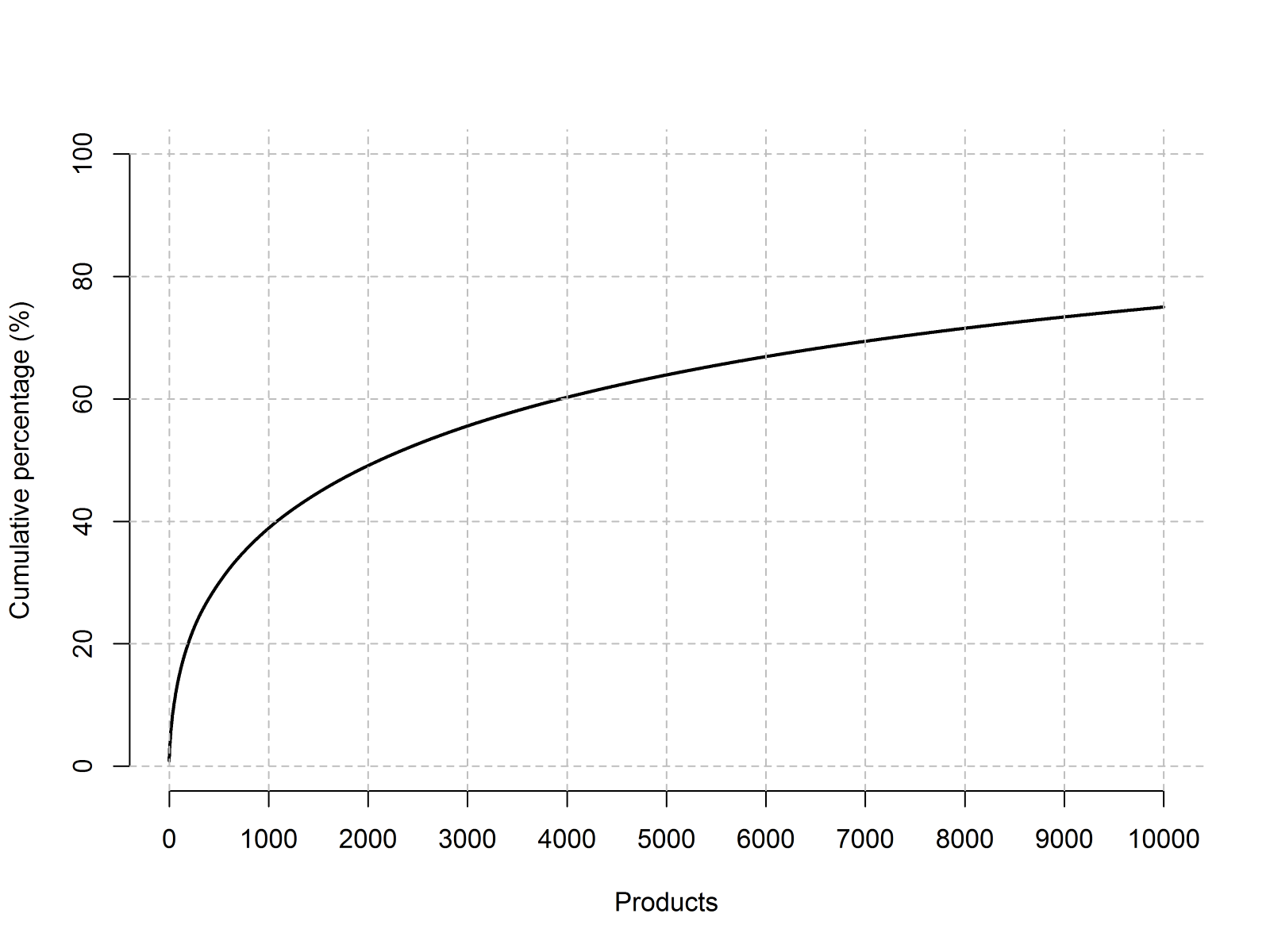


Figure S2: Cumulative percentage of total products bought that are made up by the ‘n’ most frequently bought item names
